# Supplementary material for: Inferring Resource Competition in Microbial Communities from Time Series
Source: PRX Life. Author manuscript; Available in PMC 2026 Apr 28. (PMC13120769; doi:10.1103/8gkj-rdzy)
Supplement: Supplementary Material [file NIHMS2165056-supplement-Supplementary_Material.pdf]

Inferring resource competition in microbial communities from time series — Supplemental Material

Xiaowen Chen[1], Kyle Crocker[2,3], Seppe Kuehn[2,3,4,5,*], Aleksandra M. Walczak[1,4,*] and Thierry Mora[1,4,*]
[1] *Laboratoire de Physique de l'École normale supérieure, ENS, Université PSL, CNRS, Sorbonne Université, Université Paris Cité, F-75005 Paris, France*
[2] *Department of Ecology and Evolution, The University of Chicago, Chicago, USA*
[3] *Center for the Physics of Evolving Systems, The University of Chicago, Chicago, USA*
[4] *Center for Living Systems, The University of Chicago, Chicago, USA*
[5] *National Institute for Theory and Mathematics in Biology, The University of Chicago and Northwestern University, Chicago, USA and*
[*] *Corresponding authors. The authors contributed equally to this work.*

Supplementary Information

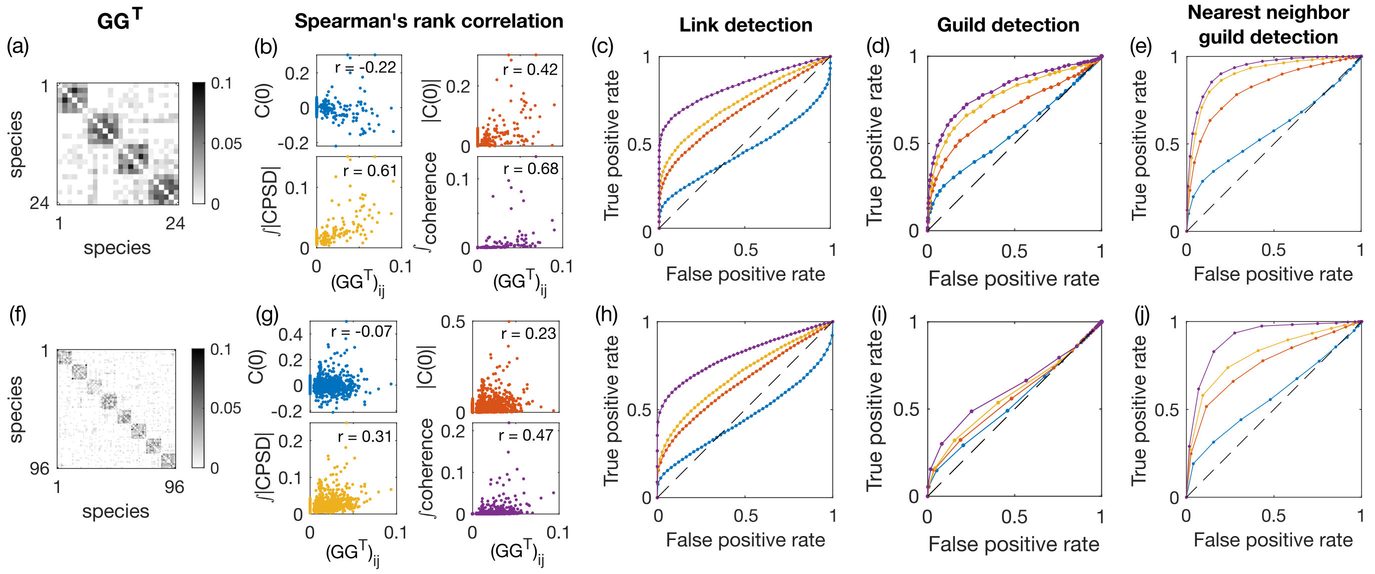

FIG. S1: **Pairwise observable analysis for the simulated ECRM ecosystem of different sizes.** Same as Fig. **??** but with $q = 0.4$.

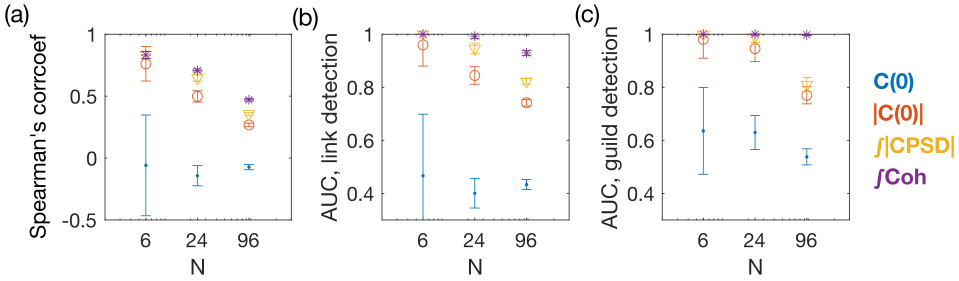

FIG. S2: **Coherence and CPSD outperforms equal-time correlation measures in recovering the resource-utilization overlap matrix $\mathbf{GG}^\intercal$ for all sizes of systems.** (a) Spearman's rank correlation coefficient between the four considered pairwise observables (equal time correlation $\mathbf{C}(0)$ and its absolute value $|\mathbf{C}(0)|$, the total magnitude of CPSD $\mathcal{P}$, and the total coherence $\mathcal{TC}$) and the elements of the $\mathbf{GG}^\intercal$ matrix, for three families of ECRM systems with $N = 6, 24, 96$ defined in Table **??**. Error bars represent standard deviation across 100 random realizations of species abundances for systems with $N = 6$ and with $N = 24$ (10 random ECRM systems each with 10 random OU environmental drives with $\omega_\alpha = 1$), and across 10 random realizations of species abundances for systems with $N = 96$ (10 random ECRM systems each with 1 random OU environmental drive with $\omega_\alpha = 1$). (b) Area under curve (AUC) for link detection for the same systems as in (a). Each AUC is computed for the same ECRM system, averaged over the ROC of all 10 random OU drives. (c) AUC for guild detection after single linkage clustering for the same systems as in (a). Guild-structure bias is $q = 0.5$.

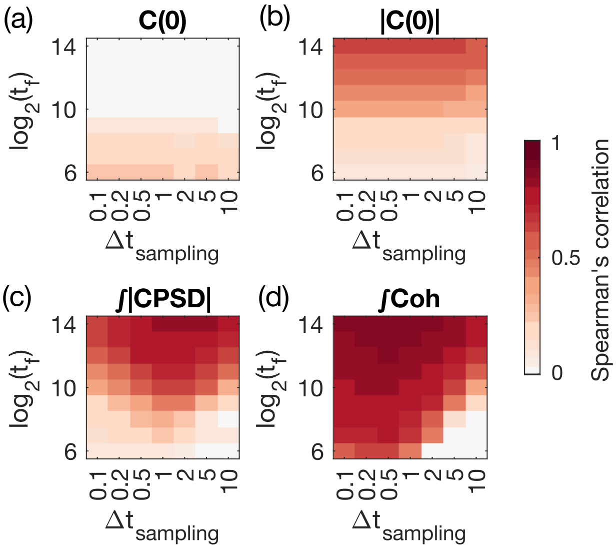

FIG. S3: **Influence of finite time-series duration and sampling rate on Spearman's correlation coefficient between the pairwise measures of species abundance and the resource utilization overlap matrix $\mathbf{GG}^\intercal$.** Spearman's correlation coefficient (colorbar) between elements of the $\mathbf{GG}^\intercal$ matrix and elements of (a) Equal-time correlation $\mathbf{C}(0)$, (b) its absolute value $|\mathbf{C}(0)|$, (c) the total absolute value of CPSD, and (d) the total coherence, as the function of sampling duration t_f and the sampling interval $\Delta t_{\text{sampling}}$. The results are averaged over 50 random realizations of the ECRMs with $N = 6$, with OU environmental drive with $\omega_\alpha = 1$ and guild-structure bias $q = 0.4$.

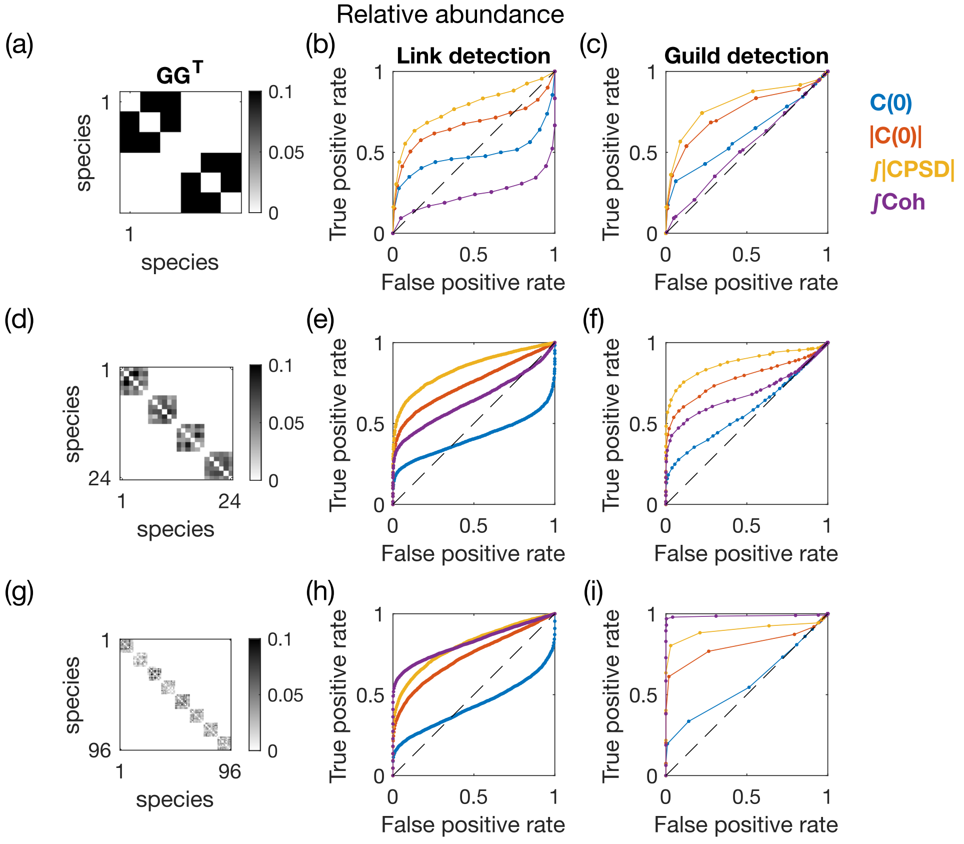

FIG. S4: **Link detection and guild detection of the resource utilization overlap matrix $\mathbf{GG}^\intercal$ using pairwise measurements of the relative abundance (instead of absolute abundance) of species concentration, for simulated ECRM ecosystems of different sizes** (Table **??**), with guild-structure bias $q = 0.5$. (a) Effective resource-utilization matrix $\mathbf{GG}^\intercal$ with $N = 6$ species, $M = 30$ resources, $k = 2$ guilds, and $q = 0.5$. (b) ROC curve of link detection for the four pairwise observables. Legend for the colors is given next to panel (c). (d) Same as (c) but for guild detection, after performing single linkage clustering on the pairwise metrics with a tunable threshold. (e-h) Same as (a-d) but for $N = 24$, $M = 120$, and $k = 4$. (i-l) Same as (a-d) but for $N = 96$, $M = 480$, and $k = 8$. For all ECRMs, the environment is driven by OU processes with the intrinsic timescale set to $\omega_\alpha = 1$. For all system sizes, the total duration of the simulated trajectory is set to $t_f = 20000$, and the sampling timestep is set to $\Delta t_{\mathrm{sampling}} = 1$.

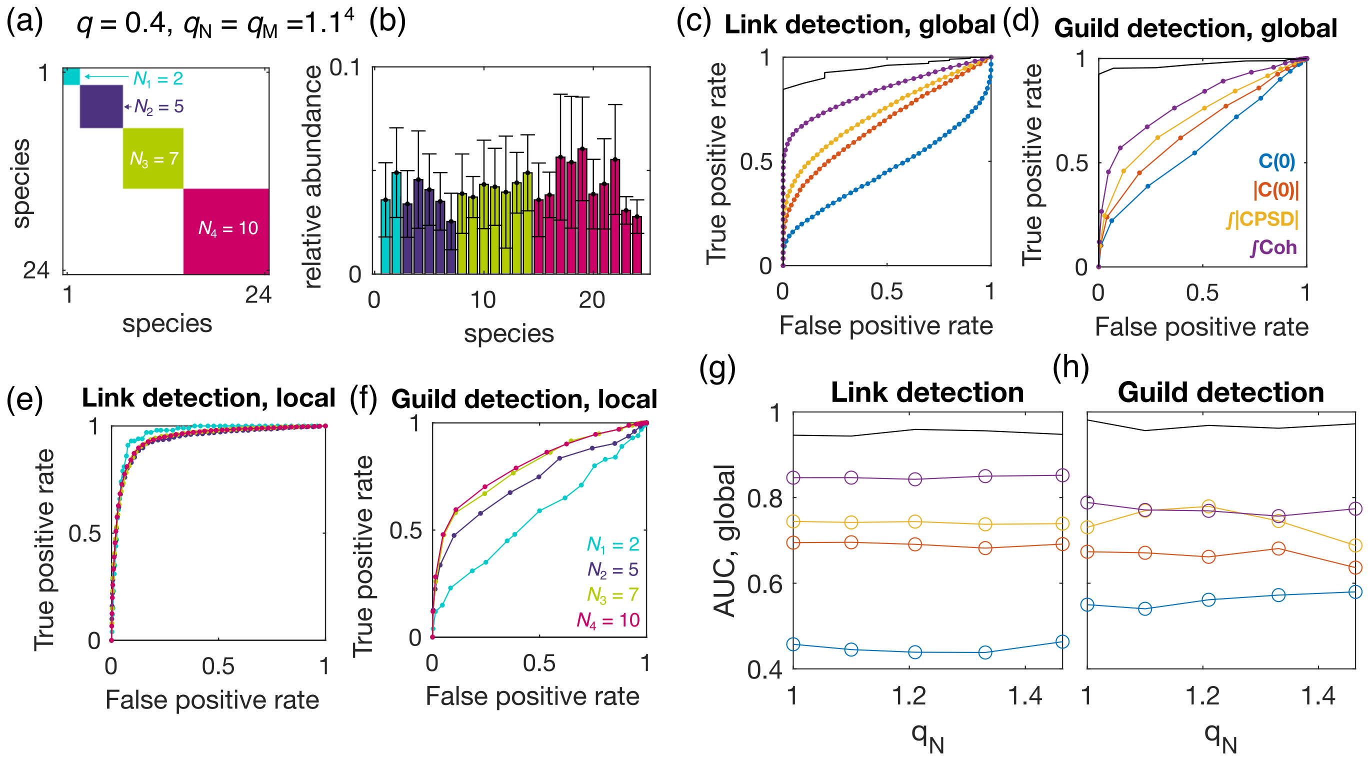

FIG. S5: **Link detection and guild detection for heterogeneous ECRM ecosystems with different guild sizes.** The guild sizes follow a geometrical series with the common ratio set by the guild-size variability q_N (see **Methods**). The average number of resources per species is tuned by $q_S = q_M/q_N$, where q_M is the common ratio of the geometrical series of the number of resources associated with each guild. For this plot, $q_S = 1$, which leads to each species being equally presented. Guild-structure bias is set to $q = 0.4$. (a) Schematics for an ECRM with $N = 24$ species, $M = 120$ resources, and $k = 4$ guilds. The guild structures at $q_N = 1.1^4$. The number of species in each guild is $N_1 = 2$, $N_2 = 5$, $N_3 = 7$, $N_4 = 10$, respectively. (b) Averaged relative abundance for each species. Color represents distinct guilds. Error bars represent standard deviation across 10 random realizations of the $\mathbf{G}\mathbf{G}^\intercal$ matrix, each with 10 random OU environmental drives with $\omega_\alpha = 1$. (c-d) ROC curve of link detection (c) and guild detection (d) for the four pairwise observables. Legend for the colors is given in panel (d). The black curve is the ROC curve if one uses the ground truth $\mathbf{G}\mathbf{G}^\intercal$ matrix as the binary classifier. Coherence remains the best predictor of the link and guild structure. (e-f) Guild-specific local detectability for link detection (e) and guild detection after single linkage clustering (f), using coherence as the binary classifier. Local detectability measures for each guild, the probability of two species correctly being identified in the guild (see detailed definition in **Methods**). Colors correspond to the four guilds with different sizes as given in panel (a). Although link detection does not depend on guild structure, guild detectability after single linkage clustering shows a considerable difference between guilds of different sizes. In particular, the smallest guild with $N_1 = 2$ becomes difficult to detect. (g-h) Link detection (g) and guild detection (h) for ECRM ecosystems with a range of guild-size variability. Here, we specify $q_N = 1, 1.1^1, \ldots, 1.1^4$. $q_S = 1$ always, such that the relative abundance for each species is even. Guild sizes are equal at $q_N = 1$, which corresponds to the discussions in the main text.

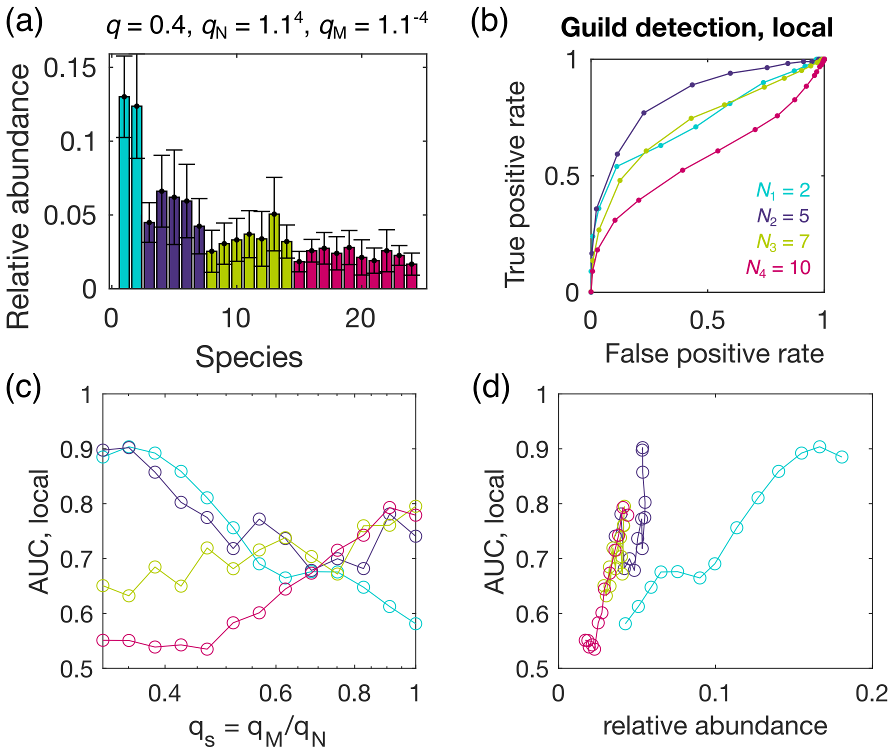

FIG. S6: **Guild detection for heterogeneous ECRM ecosystems with different guild sizes and different species abundance. Detection of small guilds is rescued by large relative abundance in those guilds.** Shown here are the results for an ECRM ecosystem with $N = 24$ species, $M = 120$ resources, $k = 4$ guilds, and guild-size variability $q_N = 1.1^4$ as exemplified by the schematics in Fig. S5(a). Averaged number of resources per species is tuned by $q_S = q_M/q_N$. (a) Relative abundance of the species in an example ECRM ecosystem, with $q_M = 1.1^{-4}$, i.e. $q_S = q_M/q_N = 1.1^{-8}$. This community is uneven. Species belonging to the smallest guild with size $N_1 = 1$ have the largest relative abundance. Colors represent distinct guilds. Error bars represent standard deviation across 10 random realization of the $\mathbf{GG}^\intercal$ matrix, each with 10 random OU environmental drives with $\omega_\alpha = 1$. (b) Guild-specific local detectability for guild detection, using coherence as the binary classifier. Curves with different colors correspond to the four different guilds. The detectability of the smallest guild with size $N_1 = 2$ (cyan) improves compared to the ECRM with the same guild sizes but even species abundance (Fig. S5)(f). (c) AUC for local guild detectability using coherence as the binary classifier (Area under the curves in panel (b)), for various species unevenness with the tuning parameter $q_S = 1.1^{-12}, 1.1^{-11}, \ldots, 1.1^{-1}, 1$. The value $q_S = 1$ corresponds to results presented in Fig. S5, where species are even. (d) AUC for local guild detectability against the mean relative abundance of all species in specific guilds. As the relative abundance increases, the AUC also increases, suggesting that guilds are more detectable if species in the guild are more abundant. Specifically, at small values of q_S, i.e when the smaller guilds have large relative abundance and the large guilds have small relative abundance, it becomes more difficult to detect the larger guilds ($N_4 = 10$ curve in panel (c)), although the AUC is still greater than 0.5. Results in (c) and (d) are averages over 10 random realizations of the ECRM systems.

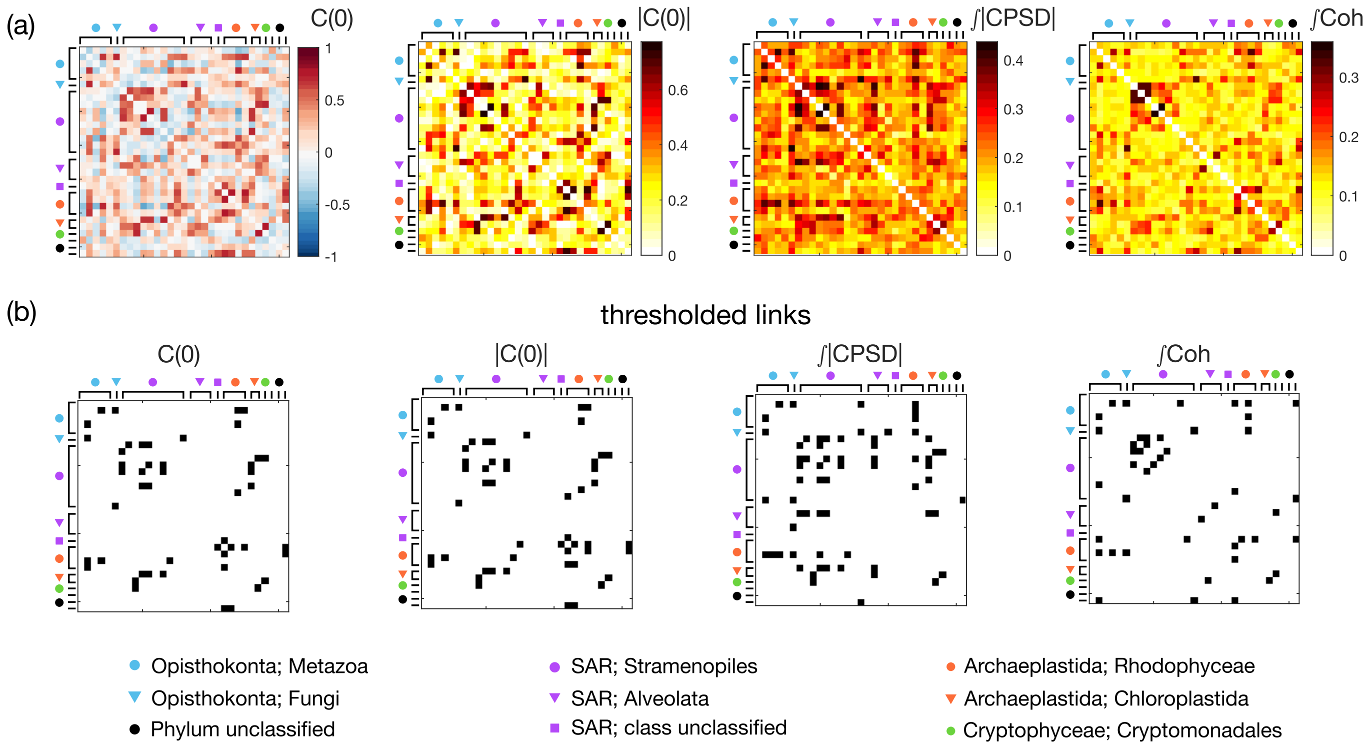

FIG. S7: **Pairwise measurements of the 93-day time series of relative abundance of eukaryotic OTUs in the Martin-Platero et al. coastal plankton dataset** (Fig. **??**, data from [**?**], downsampled to $N = 31$). (a) Equal-time correlation $\mathbf{C}(0)$ and its absolute value $|\mathbf{C}(0)|$, the total magnitude of CPSD, and the total coherence of the time-series of relative abundance of marine eukaryotic OTUs ($N = 31$). The phylum and the class of each OTU is indicated by an unique color and shape (given in legend at bottom). (b) The adjacency matrix formed by thresholding the pairwise measurements in panel a to form $k = 13$ clusters (as in Fig. **??**(c) and described in text). Black indicates a link, while white indicates no link.
